# Meteorological Observations Taken

**Published:** 1859-02

**Authors:** J. G. Westmoreland

**Affiliations:** Smithsonian Institution, January, 1859, at Atlanta, Ga.


					﻿c
Sa .	*	. .a .££££ . . . .^^fcr'a . . .£>’■£ . • •
OO I	ft . M W Z • a . . . . 02	02 . . . . •£ 02 Z . . . a a f£ <£ . . .
rH	®	Z z Z Z Z	Z Z Z	Z Z Z	Z Z Z
«-------------------------------------------------------------------------------
s
S
• •
’ ->j f5 y’ te . a a & >' it	it fe a’	. i± • . . fe fe
-	* .aa . .a .. .	. . . .zoia^ . ,a'a^^^ . .
§ ^-i	£ ct Z Z Z Z Z Z Z	Z Z Z Z	Z Z	Z Z
'•~ ©
-S
O>	____,'t . - ■_________4______________________________________________
co t—<
sS ~
S3	•	.	.	....	...
• -ww	•&:... . jt fe	. . . te fe
b	■<	aa-.a....a^^^..fe.^®®fe a" a fe fe &
S	t-’ Z Z z z z z	z z z	z z	zz
5 ?
•g^	' £
*	5
< . ©0000>00©0010'00101010000100©©0000001010
■«.£>	P.	-	c	_	C WW	H
su	Cl	V
<*9	•	. i
?J --------------------------------------------------------— “
rS — S s	1-1
q	Q , OiOOOOiOOOO©iOiOOiQiQiOOO©iOOOOOO»OOOKOiQiO I
a	*	~	r-,	r-,	_-H	£
Q	S
a	---—-----—----------------------------------------------------------- o
S • £.	.	25
-S e>	s	►?
n §	. ©lOOOOOOOOOiOiOOiOOOOiOOOOiOCOioOOOOiOO h-i
©kJ	<	~
i I	i
3	S	”	s	?
. co	a	t-h	a
<b w------------------------------------------------------------------------------ «
v . S	o
Ki	a • S3 2 22 5! 22 ®3 ® 3 £3 2 2	50 ®»® <*> « oo ct oo © © •* co ct oo co co co Z
J 3	“ f’	T ’f t ’!”+ © ’T ci a Z ? 3
X3^~	E-l	Ci
<s a	•————————— ---------------—--------------------------------------—  -
so	?	«
S	5	. C' © © Cl CT O © O M O O -C C C C © © X - O C O Cl Cl O C m m
<;	2	*	- t -f c >©T}ICO'^Tt<TtllO©©'^lOTbCO^©S^ScO^SS^S©^
_g .	g	CT______________________________
<2^§	«	x
.gy'	^^MCOCOwSjCTCTWW®“S5hm^M®M<U0m2ct®M^25®^w
n	~	~	’-J0’0:'®-*»Mco<»ooS® ocob-S2osoo r-< ct2
co TH	.	a*	OOOOOoic^OOOOOCiOOOC>OOOajo<S<~><-^(-<<-<^X^^
g	a	_	C0C0C0C0C0CTCTC0C0C0C0C0CTCTCTC0C0C0O5C0CTMWWMMro®^gg
"g	a	*	r- ct i—< .‘•2®’”°! .a?®5. ^^wwCTcoS^oowuolU^SSCTCTr-icoS
n	S	a*	ddo'oo^wooo'sooaffi'ddddoadanorioririmX
Sj	O	COCOCOCOCOCTCTCOCOCOCOCOCOCTCTCOCOCOCOCOCTCOMWMMWWMWM
g	pq S ■«. H --•>(< >O r. M	b- b- -t Cl CO Cl Cl r-H ,-< CO co CT CO >— CO CO l~ CT CO CT t~
©	a	®> ® ®) ®1 ® *® ® ®? ® ® ®1 ® ® ® ® ® I® ®> ® cc ci O> c—> j	oo <"“i ci «c ci
_g	<	CO CO CO CO CO CO Cl CO CO CO CO CO co cq ct CO CO CO CO CO CT CO CO M cow ro TO OTTO TO
,V	_ —---------------- --------------
S	Tkoir nf Mo I <X CO'tuOOb-aOOO.cCTCO^iOOb-aOOClrHCTCO^i© CO 00 Cl © rH
Vay 01 aiu. I	rH^Hrtr^'_lrtrt’-'r-,CIC4ClCI01ClClClC101COCO
				

